# Hip Joint Loading During Walking Is Associated With Cartilage Defect Severity in Young Adult Football Players With Hip/Groin Pain

**DOI:** 10.1002/jor.70219

**Published:** 2026-05-16

**Authors:** Kay M. Crossley, Yi‐Chung Lin, Anthony G. Schache, Anne J. Smith, Matthew G. King, Joshua J. Heerey, Rintje Agricola, Sharmila Majumdar, Richard B. Souza, Thomas M. Link, Benjamin F. Mentiplay, Mark J. Scholes, Adam I. Semciw, Peter R. Lawrenson, Joanne L. Kemp, Marcus G. Pandy

**Affiliations:** ^1^ La Trobe Sport and Exercise Medicine Research Centre, School of Allied Health, Human Services and Sport La Trobe University Bundoora Victoria Australia; ^2^ School of Behavioural and Health Sciences Australian Catholic University Melbourne Victoria Australia; ^3^ Sports Performance, Recovery, Injury and New Technologies (SPRINT) Research Centre Australian Catholic University Melbourne Victoria Australia; ^4^ Department of Mechanical Engineering University of Melbourne Melbourne Victoria Australia; ^5^ School of Physiotherapy and Exercise Science Curtin University Perth Western Australia Australia; ^6^ Division of Physiotherapy, School of Allied Health, Human Services and Sport La Trobe University Bundoora Victoria Australia; ^7^ Department of Orthopaedics and Sports Medicine Erasmus University Medical Centre Rotterdam The Netherlands; ^8^ Department of Radiology and Biomedical Imaging University of California San Francisco California USA; ^9^ Department of Physical Therapy and Rehabilitation Science University of California San Francisco California USA; ^10^ Division of Sport and Exercise Science, School of Allied Health, Human Services and Sport La Trobe University Bundoora Victoria Australia; ^11^ Division of Physiotherapy, School of Health and Rehabilitation Sciences University of Queensland Brisbane Queensland Australia; ^12^ Innovation and Research Centre, Community and Oral Health Metro North Health Brisbane Queensland Australia

**Keywords:** biomechanics, femoroacetabular impingement, football, hip contact force, musculoskeletal modeling, osteoarthritis

## Abstract

Understanding factors associated with early hip osteoarthritis (OA) in young active adults may facilitate hip OA prevention. We investigated the cross‐sectional association between hip contact force (HCF) during walking and cartilage defect severity, accounting for cam morphology size (alpha angle), hip pain severity, sex, age, and walking speed, in football players with hip/groin pain. One hundred and twenty‐one football players (18–50 years; 26 women) with > 6‐month hip/groin pain and no radiographic OA underwent MRI and a Dunn 45° radiograph. Outcome: Scoring Hip Osteoarthritis with MRI (SHOMRI) score for cartilage defect severity (0–20), categorized into three SHOMRI groups: (a) SHOMRI = 0; (b) SHOMRI = 1–3; and (c) SHOMRI = 4+. Movement and ground force data were captured whilst participants walked at their self‐selected pace. Musculoskeletal modeling was used to calculate HCF for one stride cycle. The exposure variable was HCF impulse (body weight times seconds [BW.s]). Differences in HCF impulse based on SHOMRI group (for the most symptomatic hip) were assessed using linear models, controlling for alpha angle, sex, age, walking speed, hip pain, and contralateral hip pain status. Resultant HCF impulse was significantly higher for participants in the 0 versus 4+ SHOMRI group (mean difference; 95%CI: 0.142BW.s; 0.028–0.256 for swing and 0.106BW.s; 0.029–0.184 for stance) and 0 versus 1–3 SHOMRI group (0.071 BW.s; 0.016–0.125 for swing). Participants with more severe cartilage defects walked with lower hip joint loading, and other variables (e.g., alpha angle size, hip pain severity, age) contributed little to this relationship. Hip joint under‐loading could be a possible target for hip OA prevention.

## 1 Background

Osteoarthritis (OA) costs global health care systems ~$24 billion annually [1], and is a leading cause of worldwide disability [2]. Those with hip OA suffer daily pain that restricts participation in work, sport/exercise, and social activities [1, 3]. It increases the risk of chronic diseases such as heart disease and diabetes [4], and cardiovascular‐related death [5]. Preventing hip OA is critical, as established OA is irreversible. We therefore need to understand how hip OA might develop or progress, focusing on young active adults, where prevention efforts would have the greatest chance of changing the OA trajectory [6].

Young active adults with hip and/or groin (hip/groin) pain may possess two characteristics that might heighten their risk of developing hip OA. Firstly, hip joint cartilage defects seen on magnetic resonance imaging (MRI), best described as early OA features, are evident in two‐thirds of young active adults with hip/groin pain [7, 8]. Cartilage defects may be reversible, or their worsening may be slowed or halted [9], but these MRI features place the individual at greater risk of end‐stage hip OA and joint replacement [10]. Secondly, young active adults with hip/groin pain also show signs of cam morphology that results from excessive bone formation at the femoral head–neck junction. Cam morphology is thought to alter joint loading patterns during daily activities, with negative effects on joint cartilage [11]. Cam morphology can increase the risk of hip OA in older adults [12], but little is known about younger, active adults with hip/groin pain.

Not all young active adults with hip cartilage defects or cam morphology will develop hip OA—other factors may play a role. Hip joint loading during everyday activities, such as walking, is integral to joint health, and either excessive load (i.e., over‐loading) [13, 14, 15], or insufficient load (i.e., under‐loading) [16, 17, 18, 19] can be deleterious. Whether altered hip joint loading in young, active adults with hip/groin pain is associated with cartilage defects or cam morphology is unknown.

This study aimed to investigate the association between hip joint contact force (HCF) during walking and cartilage defect severity, considering the influence of cam morphology size, hip pain severity, sex, age, and walking speed, in active adults with hip/groin pain.

## 2 Methods

### 2.1 Study Design and Participants (Figure 1 for Flowchart of Participants)

**Figure 1:**
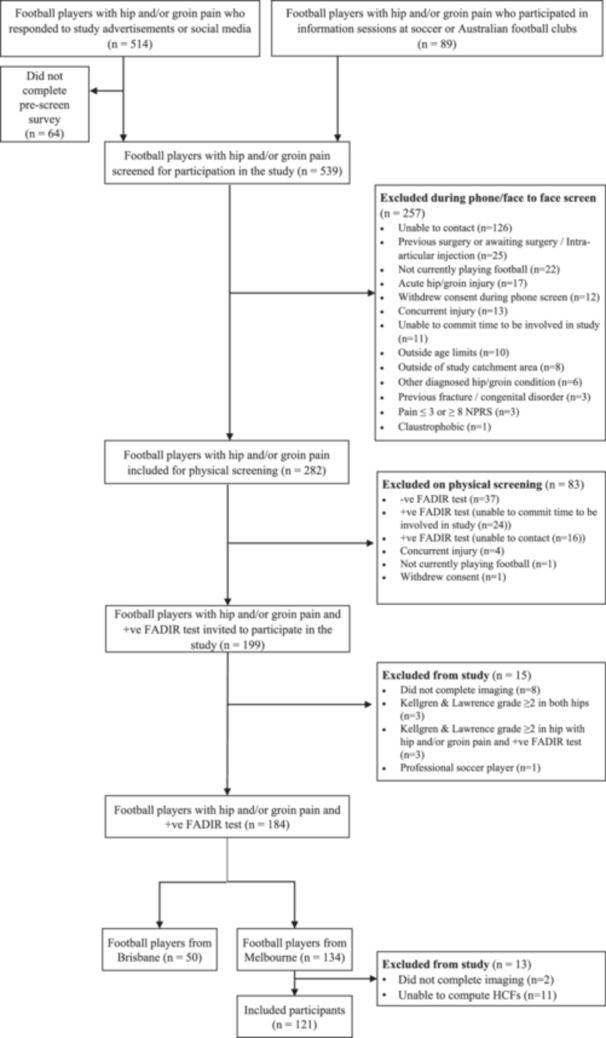
Flow chart of participants through the study. Of the 13 excluded participants, two had no MRI data, and we were unable to compute HCFs for 11 participants.

A cross‐sectional study design was adopted. Football players with hip/groin pain were recruited from baseline assessments of an ongoing longitudinal cohort study [20]. Football players were included if they met predetermined criteria (Table 1) and completed their baseline assessment at the Melbourne site (Figure 1). Recruitment was conducted via widespread social media advertisements, mailouts, and advertising through local sporting organizations. The study was approved by the La Trobe University Human Ethics Committee (HEC15‐019), and all participants provided written informed consent before participating.

**Table 1:** Study eligibility criteria.

Inclusion criteria	Exclusion criteria
− Soccer or Australian football players aged 18–50 years − Undertaking ≥ 2 sessions of football per week (training or games) − 6‐month history of gradual onset hip and/or groin pain +/− symptoms including: a. Clicking b. Locking c. Catching d. Giving way − Self‐reported pain severity of > 3 and < 8 on an 11‐point numerical rating scale^a^ with football‐specific movements, such as: a. Kicking b. Cutting/change of direction c. Squatting − Positive flexion‐adduction‐internal rotation test (FADIR)	− Self‐reported history of hip and/or groin condition or trauma, including: − Bursitis − Congenital dislocation of the hip − Osteochondritis dissecans − Fracture − Slipped capital femoral epiphysis − Subluxation or dislocation − Septic of rheumatoid arthritis − Previous hip, pelvis, or groin surgery − Kellgren and Lawrence score ≥ 2 on an anteroposterior (AP) pelvis radiograph. − Any lower limb or lumbar spine injury/complaint in the preceding 3 months. − Contraindications to radiographs or magnetic resonance imaging − Received any intra‐articular hip injection in the preceding 3 months − Unable to understand spoken or written English

^a^ Deviation from the original protocol published in [20].

### 2.2 Patient Characteristics and Patient Reported Outcome Measures

Participant characteristics (i.e., age, sex, height, mass, football code) were collected. To quantify hip‐related pain burden and quality of life, participants completed the International Hip Outcome Tool–33 (iHOT33) [21] and Copenhagen Hip and Groin Outcome Score (HAGOS) [22] (symptoms and pain subscales). These outcomes are valid, reliable and recommended for use in young and middle‐aged adults with hip/groin pain [23, 24].

### 2.3 Cartilage Defects: MRI Acquisition and Scoring

Hip MRI examinations were performed with a 3 Tesla scanner (Phillips Ingenia, The Netherlands). All participants were positioned in supine with each hip fixed in internal rotation and neutral abduction/adduction (MRI sequences are outlined in Supporting Information S1: Appendix Table B.2). A 32‐channel torso coil was placed over the hips and pelvis, with right and left hips imaged separately. Fat‐saturated coronal proton density (PD) spectral attenuated inversion recovery (SPAIR), sagittal PD SPAIR, and oblique axial PD SPAIR sequences were acquired.

One radiologist (R.S., 8 years experience), blinded to clinical and radiographic findings, completed the semi‐quantitative Scoring of Hip Osteoarthritis with MRI (SHOMRI) readings for each hip using all three imaging sequences. As cartilage loss is the hallmark feature of early hip OA [9, 25, 26] and associated hip pain [7], the cartilage defect score was the variable of interest and the only variable included in this analysis. Cartilage defects were graded from 0 to 2 (0 = no defect, 1 = partial‐thickness defect, or 2 = full thickness defect) and were scored in 4 acetabular and 6 femoral subregions (Supporting Information S1: Appendix Figure A.1), providing a total cartilage score of 0–20 for each hip. We established intra‐observer agreement (kappa 0.66, 95% CI 0.54, 0.78) for cartilage defects [7].

### 2.4 Cam Morphology: Radiograph Acquisition and Alpha Angle Measurement

A standardized Dunn 45° radiograph enabling assessment of the anterosuperior femoral head–neck junction, where maximum cam morphology is often located, was performed for each hip separately [27]. One investigator (J.J.H.), blinded to clinical findings, positioned a set of landmark points to the surface of the proximal femur (Supporting Information S1: Appendix Figure A.2) using statistical shape modeling software (ASM toolkit, Manchester University, Manchester, UK) and a shape model that was built solely with FORCe hip data [11]. Alpha angle was calculated automatically (MATLAB v 7.1.0. MathWorks Inc., Natick, Massachusetts, USA). Excellent intra‐ and inter‐observer reliability values were reported for alpha angle calculations [27].

### 2.5 Biomechanical Data Collection

Biomechanical data were collected at the La Trobe University Movement Laboratory. To allow adequate exposure of bony landmarks for marker placement, participants wore loose‐fitting running shorts (plus a crop‐top if female) and sandals (Deckers Brands, Goleta, CA). A total of 49 small (14 mm) reflective markers were affixed to the participant following previously published methods [20, 28]. Marker trajectories were collected using a 10‐camera opto‐reflective motion capture system (Vicon Motion Systems Ltd., Oxford, UK) at a frequency of 100 Hz, whereas ground reaction force (GRF) data were collected using two force plates (Advanced Mechanical Technology, Watertown, MA; Kistler Group, Winterthur, Switzerland) sampling at a frequency of 1000 Hz. Marker trajectories and raw GRF data were simultaneously recorded using Vicon Nexus v1.8.5 (Vicon Motion Systems Ltd., Oxford, UK).

A static trial was initially collected with the participant adopting a neutral stance pose. Next, participants were instructed to walk at a comfortable self‐selected speed along a 10‐meter walkway. A successful trial was defined as sequential, whole foot contact on the force plates (left then right or vice versa) at a speed ±5% of their average walking speed (determined during warmup), measured via timing gates. At least three trials were recorded per leg per participant, and the trial with the foot positioned closest to the centre of the force plate was chosen for further analysis. This decision was based on prior research showing that foot placement closer to the force plate centre improves data accuracy [29].

### 2.6 Hip Joint Contact Force Calculation

HCF was calculated bilaterally using musculoskeletal computer modeling implemented in OpenSim [30], an open‐source software platform that has been widely adopted in research and clinical communities. The process involved four main steps and was detailed in Crossley et al. [20]. First, a generic musculoskeletal model was scaled to each participant's anthropometry based on the static trial. For example, the anterior‐posterior, superior‐inferior and lateral‐medial pelvic dimensions (i.e., depth, height and width of the pelvis) were scaled linearly based on the distance between markers overlying each participant's left and right anterior superior iliac spines. Second, joint kinematics were calculated from the experimental marker trajectories by minimizing in a least‐squares sense the distances between the individual markers on the participant and the corresponding virtual markers on the model [31]. Third, individual muscle forces were calculated by solving an optimization problem using an algorithm called “computed muscle control” available in OpenSim [32]. Muscle redundancy was resolved by using a cost function that minimizes the sum of all muscle activations squared subject to the physiological constraints on the magnitude of muscle force imposed by each muscle's force–length–velocity property. Fourth, Joint Reaction Force analysis [33] in OpenSim was used to calculate HCF for each time point throughout the stride. This analysis produced the resultant force exerted at the hip joint due to all the internal (e.g., muscle forces) and external (e.g., gravity) forces acting on the model. A similar modeling approach has been used by other studies to calculate HCFs during walking in people with hip OA [17, 19].

In this study, HCF refers to the force acting on the acetabulum in the model [34]. We calculated the resultant plus the three components of the HCF (anterior–posterior, superior–inferior, lateral‐medial). All forces were expressed with respect to the femoral coordinate system and normalized to the participant's body weight (BW). For the resultant plus each component of the HCF, data were integrated over stance and swing separately to calculate the impulse (variable of interest) and expressed in units of BW.s. The impulse encapsulates both the time‐varying magnitude and duration of application of the HCF over a specified period (stance or swing). We analyzed the impulse of the HCF rather than its magnitude at discrete time points because the impulse represents the integrated effect of the HCF over time and is likely to be less sensitive to data filtering procedures.

### 2.7 Data Management and Analysis

Whilst we recorded imaging and HCF data bilaterally, only the symptomatic hip was evaluated. For participants with bilateral symptoms, the hip that caused the most trouble (as per iHOT33 questionnaire) was used for analysis. Linearity of associations between total cartilage SHOMRI scores (exposure) with HCF impulse (outcome) was initially explored via scatterplots (Supporting Information S1: Appendix Figure A.3). To appropriately capture the presence of non‐linearity in the relationship between the outcome variables and total cartilage SHOMRI scores, and the right skew of the distribution, the total SHOMRI score was categorized into three SHOMRI groups: (a) SHOMRI = 0; (b) SHOMRI = 1–3; and (c) SHOMRI = 4+. This categorization is broadly consistent with recommendations for reporting semi‐quantitative knee MRI gradings [35].

Demographic data and patient‐reported outcome measures were explored for normality using the Shapiro–Wilk test and visual inspection of boxplots and reported as means and standard deviations or medians and interquartile ranges as appropriate. Differences between SHOMRI groups (exposure) were assessed using linear regression models for the impulse of the resultant and the three components of the HCF (outcome). For each HCF impulse (resultant; anterior‐posterior; superior‐inferior; lateral‐medial) and phase of the stride cycle during walking (stance; swing), SHOMRI group (categorical exposure variable) and potential confounders of alpha angle, hip pain severity (iHOT item Q16: Overall how much pain do you have in your hip/groin? 0 = extreme pain, visual analog scale: 100 = no pain at all), sex, contralateral hip pain status, age, and walking speed were entered into the model with the *F*‐value and *p* value reported for all models. Post hoc pairwise comparisons between SHOMRI groups controlling for alpha angle, hip pain severity (Q16 on the iHOT33), sex, contralateral hip pain status (binary outcome of symptomatic vs. asymptomatic), age, and walking speed were performed for all models and reported as adjusted mean differences with 95% confidence intervals, *p* values and Cohen's *d* effect sizes (interpreted as 0.2 = small; 0.5 = moderate; ≥ 0.8 large effects). All statistical analysis was conducted in R (R Foundation for Statistical Computing) using the “car” and “emmeans” packages, with the “performance” package used to confirm linear model assumptions.

### 2.8 Patient and Public Involvement

No patient or public involvement was included in the design, conduct, or reporting of this study.

### 2.9 Protocol Deviation

The longitudinal cohort study [20] was conducted at two different sites within Australia: Melbourne and Brisbane. However, only the data recorded from the 134 participants at the Melbourne site are included in the present study due to unforeseen inconsistencies in laboratory configurations between the two sites (note: there were no meaningful differences in participant demographics between the sites—Supporting Information S1: Appendix Table B.1).

## 3 Results

For this study, 121 young adult football players (26 women; average age 26.7 (standard deviation 5.7) years; height 1.79 (0.08) m, mass 78.5 (12.3) kg) had complete data collection and analyses (Figure 1). Participant characteristics are in Table 2. Most participants had either SHOMRI = 0 (*n* = 53/44%) or SHOMRI = 1–3 (*n* = 51/42%) cartilage defects, whereas only 17/14% had SHOMRI = 4+ [range 4–7] cartilage defects (Supporting Information S1: Appendix Figure A.3). Two‐thirds of the cohort had bilateral symptoms (*n* = 80/66%). The temporal patterns and magnitudes of the time‐normalized pelvis and lower‐limb joint angles and moments for SHOMRI groups are presented in Supporting Information S1: Appendix Figures A.4, A.5.

**Table 2:** Characteristics of included participants (*n* = 121) across the SHOMRI groups.

	SHOMRI = 0	SHOMRI = 1–3	SHOMRI = 4+
Demographic characteristics			
*n* (women/men)	53 (16/37)	51 (7/44)	17 (3/14)
Age, y^a^	26.0 [6.0]	25.0 [5.4]	28.0 [8.4]
Height, m	1.78 (0.08)	1.80 (0.08)	1.78 (0.08)
Mass, kg	75.3 (12.2)	80.4 (12.1)	82.7 (12.2)
Alpha angle (°)	65.8 (15.2)	73.5 (15.8)	65.8 (11.6)
Walking speed (m/s)	1.47 (0.15)	1.45 (0.13)	1.46 (0.11)
Sport			
Soccer	*n* = 18, 34%	*n* = 20, 39%	*n* = 10, 59%
Australian football	*n* = 35, 66%	*n* = 31, 61%	*n* = 7, 41%
Training/competition (per week), %			
2–3 sessions	*n* = 48, 91%	*n* = 46, 90%	*n* = 14, 82%
≥ 4 sessions	*n* = 5, 9%	*n* = 5, 10%	*n* = 3, 18%
Duration of symptoms, months^a^	27 [18, 60]^b^	24 [16, 48]	36 [18, 102]
Radiographic measures			
Kellgren and Lawrence grade, %			
Grade 0	*n* = 53, 100%	*n* = 46, 90%	*n* = 16, 94%
Grade 1	*n* = 0, 0%	*n* = 5, 10%	*n* = 1, 6%
Patient‐reported outcome measures			
iHOT33‐total	55.7 (16.5)	57.5 (17.2)	53.6 (15.8)
iHOT33 Q16	51.8 (21.9)	51.4 (20.6)	54.5 (26.6)
HAGOS–symptoms	53.5 (13.8)	54.9 (14.0)	55.7 (15.9)
HAGOS–pain	63.9 (13.7)	64.5 (16.1)	60.4 (18.9)

*Note:* Data reported as means and standard deviations unless indicated.

iHOT‐33 = International hip outcome tool 33 (/100) 0 = worse possible score, 100 = best possible score; iHOT 33 Q16: overall, how much pain do you have in your hip/groin? (/100) 0 = extreme pain, 100 = no pain at all. HAGOS = Copenhagen hip and groin outcome score (/100) 0 = worse possible score, 100 = best possible score.

Abbreviation: m/s = meters per second.

^a^ Median and interquartile range.

^b^ *n* = 52 participants.

### 3.1 Resultant Hip Joint Contact Force Impulse

**Figure 2:**
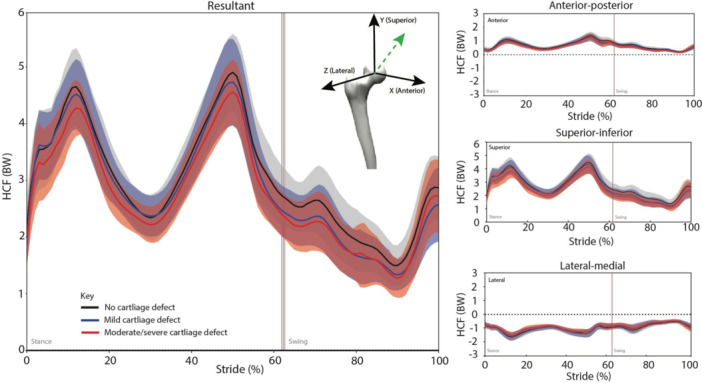
Hip joint contact force during walking for participants categorized into three SHOMRI groups: (i) SHOMRI = 0 (black solid line); (ii) SHOMRI = 1–3 (blue solid line); and (iii) SHOMRI = 4+ (solid red line). In each panel, data are displayed for a single full stride cycle for the reference/test limb from ipsilateral foot strike (0%) to the next ipsilateral foot‐strike (100%), with the thin vertical lines indicating ipsilateral toe‐off (~60%). The resultant HCF is displayed in the left panel, whereas three components of the HCF are displayed in the right panels. In all panels, the HCF represented the force directly applied to the acetabulum by the femoral head. The broken green arrow in the inset (left panel) represents the resultant HCF.

The resultant HCF (Figure 2) displayed a biphasic pattern during stance. Linear regression revealed differences between SHOMRI groups for the resultant HCF impulse in swing, but not stance phase, adjusting for covariates (alpha angle, hip pain severity, sex, contralateral hip pain status age, walking speed) (Figure 2; Table 3; Full model output: Supporting Information S1: Appendix Tables B.3,B.4). Post‐hoc analyses revealed a larger resultant HCF impulse for participants in the SHOMRI = 0 group, compared to those in the SHOMRI = 4+ group (mean difference; 95%CI: 0.142; 0.028–0.256 BW.s) during stance, and compared to those in the SHOMRI = 1–3 group (0.071; 0.016–0.125 BW.s) and SHOMRI = 4+ group (0.106; 0.029–0.184 BW.s) during swing (Figure 2; Table 3; Full model output: Supporting Information S1: Appendix Tables B.3,B.4). These relationships had small to moderate effect sizes (*d* = 0.30–0.40).

**Table 3:** Results of linear models and post hoc comparisons comparing hip joint contact force during the stance and swing phase of walking.

Plane	Phase	Linear model results: *F*‐value, *p*‐value	Post‐hoc comparisons: #Adjusted mean difference (95% CI), P‐value, Cohen's *d*		
			SHOMRI = 0 vs. SHOMRI = 1–3	SHOMRI = 0 vs. SHOMRI = 4+	SHOMRI = 1–3 vs. SHOMRI = 4+
Resultant	Stance	*F* = 3.0643, *p* = 0.051	0.042 (−0.038 to 0.122) *p* = 0.298, *d* = 0.39	0.142 (0.028–0.256) *p* = 0.015**, *d* = 0.30	0.010 (−0.016 to 0.216) *p* = 0.091, *d* = 0.11
	Swing	*F* = 5.257, *p* = 0.007*	0.071 (0.016–0.125) *p* = 0.012**, *d* = 0.37	0.106 (0.029–0.184) *p* = 0.008**, *d* = 0.40	0.036 (−0.043 to 0.114) *p* = 0.370, *d* = 0.13
Anterior‐posterior	Stance	*F* = 3.694, *p* = 0.028*	0.028 (0.002–0.054) *p* = 0.033**, *d* = 0.21	0.042 (0.005–0.079) *p* = 0.025**, *d* = 0.27	0.014 (−0.023 to 0.052) *p* = 0.452, *d* = 0.09
	Swing	*F* = 3.911, *p* = 0.023*	0.016 (0.003–0.030) *p* = 0.021**, *d* = 0.23	0.022 (0.002–0.042) *p* = 0.028**, *d* = 0.27	0.006 (−0.014 to 0.026) *p* = 0.566, *d* = 0.07
Superior‐inferior	Stance	*F* = 2.993, *p* = 0.054	0.037 (−0.036 to 0.111)*p* = 0.315, *d* = 0.10	0.129 (0.024–0.234) *p* = 0.016**, *d* = 0.29	0.091 (−0.015 to 0.198) *p* = 0.091, *d* = 0.21
	Swing	*F* = 5.916, *p* = 0.004*	0.067 (0.018–0.116) *p* = 0.007**, *d* = 0.27	0.101 (0.031–0.170) *p* = 0.005**, *d* = 0.34	0.034 (−0.037 to 0.104) *p* = 0.344, *d* = 0.12
Lateral‐medial	Stance	*F* = 1.493, *p* = 0.229	0.006 (−0.029 to 0.041) *p* = 0.738, *d* = 0.03	0.043 (−0.007 to 0.93) *p* = 0.090, *d* = 0.20	0.037 (−0.013 to 0.088) *p* = 0.149, *d* = 0.18
	Swing	*F* = 2.182, *p* = 0.118	0.021 (−0.003 to 0.043) *p* = 0.080, *d* = 0.17	0.027 (−0.006 to 0.060) *p* = 0.104, *d* = 0.20	0.007 (−0.027 to 0.040) *p* = 0.689, *d* = 0.05

*Note:* # All data reported as means (standard deviation) in BW.s. Linear models and post‐hoc comparisons adjusted for alpha angle, sex, age, walking speed, hip pain, and contralateral hip status.

Abbreviations: CI, confidence interval; SD, standard deviation.

* linear model *p* < 0.05.

** post hoc comparison *p* < 0.05.

### 3.2 Anterior–Posterior Hip Joint Contact Force Impulse

Linear regression revealed differences between SHOMRI groups for the anterior HCF impulse in stance and swing phases, adjusting for covariates (Figure 2; Table 3; Full model output: Supporting Information S1: Appendix Tables B.3,B.4). Post‐hoc analyses revealed that the anterior HCF impulse was greater for participants in the SHOMRI = 0 group, compared to those in the SHOMRI = 1–3 group (0.028; 0.002–0.054 BW.s) and SHOMRI = 4+ group (0.042; 0.005–0.079 BW.s) during stance, and compared to those in the SHOMRI = 1–3 group (0.016; 0.003–0.030 BW.s) and SHOMRI = 4+ group (0.022; 0.002–0.042 BW.s) during swing (Figure 2; Table 3; Full model output: Supporting Information S1: Appendix Tables B.3,B.4). These relationships had small effect sizes (*d* = 0.21–0.27).

### 3.3 Superior–Inferior Hip Joint Contact Force Impulse

Linear regression revealed differences between SHOMRI groups for the superior HCF impulse in swing, but not stance phase, adjusting for covariates (Figure 2; Table 3; Full model output: Supporting Information S1: Appendix Tables B.3,B.4). Post‐hoc analyses revealed that the superior HCF impulse was larger for participants in the SHOMRI = 0 group, compared to those in the SHOMRI = 4+ group (0.129; 0.024–0.234 BW.s) during stance, and compared to those in the SHOMRI = 1–3 group (0.067; 0.018–0.116 BW.s) and SHOMRI = 4+ group (0.101; 0.031–0.170 BW.s) during swing (Figure 2; Table 3; Full model output: Supporting Information S1: Appendix Tables B.3,B.4). These relationships had small to moderate effect sizes (*d* = 0.27–0.34).

### 3.4 Lateral–Medial Hip Joint Contact Force Impulse

Linear regression revealed no significant associations between the cartilage defect severity groups and the medial HCF impulse in stance or swing phases (Figure 2; Table 3; Full model output: Supporting Information S1: Appendix Tables B.3,B.4).

## 4 Discussion

In 121 young (average age 27 (standard deviation 6) years) active adults (football players) with persistent hip/groin pain and no signs of radiographic hip OA, a lower impulse of the HCF during both the stance and swing phases of the walking cycle was associated with a more severe cartilage defect score—which seems independent of cam morphology and hip pain severity. Nevertheless, because the effect sizes for our statistically significant comparisons ranged from 0.21 to 0.40, we acknowledge that the strength of the relationship between load and structure at the hip was modest at best. Our findings have potential to impact hip OA prevention as we evaluated young adults with, or at risk of, early hip OA features—those for whom prevention strategies might be most effective. This cross‐sectional study cannot illuminate the causal relationship between HCF magnitude and cartilage defect severity; however, it provides valuable insights regarding the relationship between load and structure at the hip, considering multiple potential confounders, including cam morphology size (alpha angle), sex, age, walking speed and hip pain severity. Our findings, alongside those from other studies of older people with radiographic hip OA [16, 36], point to a potential link between under‐loading and worsening hip OA.

Cam morphology in adults > 45 years‐old is a risk factor for incident and end‐stage hip OA [12]. Much less is known about younger adults with, or at risk of, early hip OA features. Our prior work involving the same cohort identified an association between larger cam morphology (higher alpha angle) and more substantial cartilage loss [11]. Whilst we do not know if cam morphology in young active adults increases the risk of incident or worsening hip OA, it is likely that additional factors influence this potential relationship. Cam morphology is thought to precipitate hip joint damage and OA through mechanical abutment with the acetabulum during activities that involve large hip movements, and in the presence of hip symptoms, this entity is termed femoroacetabular impingement syndrome [37]. In people with hip/groin pain, it is possible that those with larger cam morphology might adjust their walking patterns to avoid bony impingement, which could result in lower HCFs. Whilst our results did not support this theory (see Supporting Information S1: Appendix Table B.4), it is possible that alpha angle may influence other biomechanical variables (e.g., cartilage stress or strain). It is also possible that a larger cam might affect movement patterns and HCFs in activities requiring greater ranges of motion than walking (e.g., sprinting/change of direction/lunging).

The lower HCF impulses during walking for the SHOMRI = 4+ group appeared to be due to the combined effect of some small differences in pelvis and hip joint angles, as well as hip joint moments. For example, participants in the SHOMRI = 4+ group displayed less frontal plane angular excursion at the pelvis and hip joint across the stride cycle (Supporting Information S1: Appendix Figure A.4). They also displayed a lower net internal hip extensor moment during early stance (~10% of the stride cycle) and a lower net internal hip flexor moment during late stance (~50% of stride cycle) (Supporting Information S1: Appendix Figure A.5). It is possible that our adult football players with more severe cartilage defects adopted pain avoidance walking patterns—when we are injured or in pain, we reduce the load on our injured or painful side as a protective and instinctive response. These alterations (e.g., limping) may be subtle, and undetectable to the eye, requiring more sophisticated laboratory‐based biomechanical testing to detect. Nevertheless, even though the hip/groin pain burden was substantial (mean scores ranged from 51–65/100), it was not different between the SHOMRI groups, and hip pain severity did not influence the association between the HCF impulse and cartilage defect severity (Supporting Information S1: Appendix Table B.3). Therefore, in active adults with (or at risk of) early hip OA, the lower HCF impulse seen in those with more severe SHOMRI cartilage defects does not appear to be primarily attributable to greater hip pain.

Optimal joint loading is integral to long‐term cartilage health [14], but excessive or insufficient joint loading may lead to cartilage breakdown [38]. For people with hip OA, or femoroacetabular impingement syndrome, studies investigating HCFs during walking reveal a pattern of under‐loading [16, 17, 19, 39]. Our results from young active adults not awaiting surgery indicate that under‐loading may be relevant to the early stages of hip OA, that is, the presence of cartilage defects prior to radiographic joint changes. It is possible that over‐loading plays a role in initiating early OA, but once pathology is triggered then persistent under‐loading may be deleterious. Hip joints with early OA features may not have the capacity to counter the negative consequences of subtle but persistent under‐loading, and associated muscle atrophy.

To change the course of hip OA, we need to identify modifiable factors that might modulate the trajectory, ideally in those without established (irreversible) disease [6]. Our findings suggest that hip under‐loading might be a target for interventions designed to halt or slow the development or progression of hip OA. Optimizing joint loading, even a subtle increase, has biological plausibility as a prevention strategy and might underpin some of the positive benefits seen with exercise‐therapy [40, 41]. But before recommendations for potential prevention strategies can be made—we need longitudinal cohort studies (and replication studies) to clearly identify prognostic groups and target variables, and clinical trials of preventative strategies.

If joint under‐loading contributes to hip OA worsening, then strategies to optimize HCFs might offer a solution. Possible interventions include hip muscle strengthening—stronger hip muscles enhance joint stability, co‐ordination and function, and provide joint compression during dynamic tasks [34, 42]. Hip joint loading may also be modulated by movement retraining strategies [41]. But “one size fits all” treatments are likely inappropriate for the variety of young active people at risk of hip OA with differing risk factors— recent hip OA studies suggest that better outcomes may be gained from personalized approaches [41, 43].

Early hip OA features, such as cartilage defects and labral tears, in young active adults, are common [7, 8, 44]. In our cohort of 182 young active adults [20] with hip/groin pain but no radiographic OA, 50%–70% have early OA features (cartilage defects and labral tears) on MRI [7]. Whilst highly prevalent, these OA features are low‐grade, and we do not know which factors might contribute to their worsening. At the knee, early OA signs are evident in young active adults following serious knee injury (e.g., anterior cruciate ligament, meniscal tear, patellar dislocation) [45] and termed “post‐traumatic knee OA”. In post‐traumatic knee OA, under‐loading has also been revealed, and evidence, albeit from small studies, indicates that under‐loading may be a feature of worsening knee OA in these young adults with heightened OA risk [46, 47, 48]. Our current study highlights that “post‐traumatic OA” might occur at the hip—not resulting from a high‐energy traumatic injury but following repetitive low‐energy trauma, where consonant features of under‐loading are observed in young adults with early signs of OA.

### 4.1 Limitations

Our study has some limitations that can influence our findings' interpretation. First, demographic differences in age and body weight were present but small (< 10%) and were included as potential confounders in our models. Second, 13 participants were not included, as 2 had no MRI data, and we were unable to compute HCFs for 11 participants. The characteristics of these 13 participants (average age of 27 (standard deviation 5.5) years; height of 1.77 (0.11) m; body mass of 78.5 (17.6.3) kg) were similar to those of the 121 participants with complete data. Third, we used musculoskeletal modeling in the present study to calculate HCFs, an approach that is dependent upon various input parameters and assumptions [49]. Our HCFs were similar in profile but larger in magnitude when compared to in vivo data measured via instrumented prostheses [50, 51]. All musculoskeletal models were linearly scaled based on participant anthropometry. Whilst we found good agreement in hip joint moments obtained from inverse dynamics versus model (see Supporting Information S1: Appendix Figure A.6), we recognize that linear scaling does not account for individual variations in muscle strength or morphology. Furthermore, by not incorporating muscle electromyography activity into our models, we did not account for any variability in motor control that may have existed between SHOMRI groups. Fourth, we only analysed one trial per participant; however, a sensitivity analysis in a subset of participants (see Supporting Information S1: Appendix Figure A.7) showed within‐participant inter‐trial variability to be small. Fifth, the internal HCF reflects “global” hip joint loading. It cannot be attributed to specific sites or anatomical structures within the hip joint [52]. Despite our findings, it is possible that those with more severe cartilage defects had localized areas within the hip that experienced higher stresses. Sixth, by choosing to evaluate only one hip in those with bilateral symptoms, we cannot rule out the possibility that the contra‐lateral hip might have influenced the relationship between HCF and cartilage defect severity for the most symptomatic hip—but we did account for contralateral hip pain status in our data analysis (Supporting Information S1: Appendix Table B.4). Seventh, the interplay between joint loading and structural degradation is likely influenced by exposure. Walking is a common task, but relative to other tasks such as running and jumping, it exposes the body (and hence the hip) to much lower average loads—and it is possible that our results might be task specific. It is also important to note that our study does not establish causation. Longitudinal cohort studies (and replication studies) and clinical trials are now urgently needed. Eighth, men and women have different risk profiles for hip OA and have known differences in biomechanics during walking. While the relationship between cartilage defects, cam morphology, hip pain and hip joint loading may be sex specific [28, 53], we did not have sufficient women in this cohort to explore sex as an effect modifier. Finally, because we did not include a control group in the present study, we do not know if our young adult football players with hip/groin pain walked with lower HCFs compared to age‐ and activity‐match asymptomatic individuals.

## 5 Conclusions

Early hip OA features have the potential to be reversed or halted [9], but if we do nothing, these young active adults risk developing end‐stage hip OA and joint replacement. We found that participants with more severe cartilage defects demonstrated a lower HCF impulse during walking, and that other variables (e.g., alpha angle size, hip pain severity, age) contributed little to this relationship. Hip joint under‐loading is a modifiable feature that may contribute to hip OA development, and is a possible target for hip OA prevention.

## Author Contributions

K.M.C., M.G.P., S.M., A.J.S., and R.A. initiated the study and acquired the funding. A.G.S., A.I.S., R.B.S., T.M.L., J.L.K., and B.F.M. refined methodology, supervised doctoral candidates and assisted with data collection or analyses. M.G.K., J.J.H., M.J.S. and P.R.L. were doctoral candidates who collected all the data for this project. Y.C.L. performed all of the modeling under the supervision of M.G.P. S.M., T.M.L., and R.B.S. oversaw all the imaging analyses. M.G.K. and A.J.S. completed the data analyses. All authors contributed to data interpretation and critically reviewed and revised the manuscript. All authors approved the final version of the manuscript. K.M.C. acts as the guarantor and accepts full responsibility for the finished work and the conduct of the study, had access to the data and controlled the decision to publish.

## Supporting information

Supporting File.

## Data Availability

The data that support the findings of this study are available on request from the corresponding author. The data are not publicly available due to privacy or ethical restrictions.

## References

[1] Australian Bureau of Statistics, “National Health Survey,” Canberra: ABS, 2009.

[2] L. B. Murphy , C. G. Helmick , T. A. Schwartz , et al., “One in Four People May Develop Symptomatic Hip Osteoarthritis in His or Her Lifetime,” Osteoarthritis and Cartilage 18, no. 11 (2010): 1372–1379. 20713163 10.1016/j.joca.2010.08.005 PMC2998063

[3] M. Cross , E. Smith , D. Hoy , et al., “The Global Burden of Hip and Knee Osteoarthritis: Estimates From the Global Burden of Disease 2010 Study,” Annals of the Rheumatic Diseases 73, no. 7 (2014): 1323–1330. 24553908 10.1136/annrheumdis-2013-204763

[4] A. Williams , S. J. Kamper , J. H. Wiggers , et al., “Musculoskeletal Conditions May Increase the Risk of Chronic Disease: A Systematic Review and Meta‐Analysis of Cohort Studies,” BMC Medicine 16, no. 1 (2018): 167. 30249247 10.1186/s12916-018-1151-2 PMC6154805

[5] N. Veronese , E. Cereda , S. Maggi , et al., “Osteoarthritis and Mortality: A Prospective Cohort Study and Systematic Review With Meta‐Analysis,” Seminars in Arthritis and Rheumatism 46, no. 2 (2016): 160–167. 27179749 10.1016/j.semarthrit.2016.04.002

[6] L. S. Lohmander and E. M. Roos , “Disease Modification in OA—Will We Ever Get There?,” Nature Reviews Rheumatology 15, no. 3 (2019): 133–135. 10.1038/s41584-019-0174-1 30733580

[7] J. J. Heerey , R. Srinivasan , R. Agricola , et al., “Prevalence of Early Hip OA Features on MRI in High‐Impact Athletes. The Femoroacetabular Impingement and Hip Osteoarthritis Cohort (FORCe) Study,” Osteoarthritis and Cartilage 29, no. 3 (2021): 323–334. 33387651 10.1016/j.joca.2020.12.013 PMC8900484

[8] J. J. Heerey , J. L. Kemp , A. B. Mosler , et al., “What Is the Prevalence of Imaging‐Defined Intra‐Articular Hip Pathologies in People With and Without Pain? A Systematic Review and Meta‐Analysis,” British Journal of Sports Medicine 52, no. 9 (2018): 581–593. 29540366 10.1136/bjsports-2017-098264

[9] T. C. Pollard , S. E. Gwilym , and A. J. Carr , “The Assessment of Early Osteoarthritis,” Journal of Bone and Joint Surgery. British Volume 90, no. 4 (2008): 411–421. 18378911 10.1302/0301-620X.90B4.20284

[10] M. Kowalczuk , M. Yeung , N. Simunovic , and O. R. Ayeni , “Does Femoroacetabular Impingement Contribute to the Development of Hip Osteoarthritis? A Systematic Review,” Sports Medicine and Arthroscopy Review 23, no. 4 (2015): 174. 26524551 10.1097/JSA.0000000000000091

[11] J. Heerey , J. Kemp , R. Agricola , et al., “Cam Morphology Is Associated With MRI‐Defined Cartilage Defects and Labral Tears: A Case‐Control Study of 237 Young Adult Football Players With and Without Hip and Groin Pain,” BMJ Open Sport & Exercise Medicine 7, no. 4 (2021): e001199. 10.1136/bmjsem-2021-001199 PMC8679114 34987861

[12] R. Agricola , M. M. A. van Buuren , J. L. Kemp , H. Weinans , J. Runhaar , and S. M. A. Bierma‐Zeinstra , “Femoroacetabular Impingement Syndrome in Middle‐Aged Individuals Is Strongly Associated With the Development of Hip Osteoarthritis Within 10‐Year Follow‐Up: A Prospective Cohort Study (CHECK),” British Journal of Sports Medicine 58, no. 18 (2024): 1061–1067. 39074968 10.1136/bjsports-2024-108222 PMC11420741

[13] N. D'Souza , J. Charlton , J. Grayson , et al., “Are Biomechanics During Gait Associated With the Structural Disease Onset and Progression of Lower Limb Osteoarthritis? A Systematic Review and Meta‐Analysis,” Osteoarthritis and Cartilage 30, no. 3 (2022): 381–394. 34757028 10.1016/j.joca.2021.10.010

[14] D. J. Hunter and S. Bierma‐Zeinstra , “Osteoarthritis,” Lancet 393, no. 10182 (2019): 1745–1759. 31034380 10.1016/S0140-6736(19)30417-9

[15] T. C. Liao , M. A. Samaan , T. Popovic , et al., “Abnormal Joint Loading During Gait in Persons With Hip Osteoarthritis Is Associated With Symptoms and Cartilage Lesions,” Journal of Orthopaedic & Sports Physical Therapy 49, no. 12 (2019): 917–924. 31610757 10.2519/jospt.2019.8945 PMC7935417

[16] L. E. Diamond , H. X. Hoang , R. S. Barrett , et al., “Individuals With Mild‐to‐Moderate Hip Osteoarthritis Walk With Lower Hip Joint Contact Forces Despite Higher Levels of Muscle Co‐Contraction Compared to Healthy Individuals,” Osteoarthritis and Cartilage 28, no. 7 (2020): 924–931. 32360739 10.1016/j.joca.2020.04.008

[17] S. Van Rossom , J. Emmerzaal , R. van der Straaten , et al., “The Biomechanical Fingerprint of Hip and Knee Osteoarthritis Patients During Activities of Daily Living,” Clinical Biomechanics 101 (2023): 105858. 36525720 10.1016/j.clinbiomech.2022.105858

[18] J. Fitzgerald , J. Endicott , U. Hansen , and C. Janowitz , “Articular Cartilage and Sternal Fibrocartilage Respond Differently to Extended Microgravity,” NPJ Microgravity 5, no. 1 (2019): 3. 30793021 10.1038/s41526-019-0063-6 PMC6379395

[19] C. A. G. Meyer , M. Wesseling , K. Corten , et al., “Hip Movement Pathomechanics of Patients With Hip Osteoarthritis Aim at Reducing Hip Joint Loading on the Osteoarthritic Side,” Gait & Posture 59 (2018): 11–17. 28968547 10.1016/j.gaitpost.2017.09.020

[20] K. M. Crossley , M. G. Pandy , S. Majumdar , et al., “Femoroacetabular Impingement and Hip Osteoarthritis Cohort (Force): Protocol for a Prospective Study,” Journal of Physiotherapy 64, no. 1 (2018): 55. 29289588 10.1016/j.jphys.2017.10.004

[21] N. G. H. Mohtadi , D. R. Griffin , M. E. Pedersen , et al., “The Development and Validation of a Self‐Administered Quality‐of‐Life Outcome Measure for Young, Active Patients With Symptomatic Hip Disease: The International Hip Outcome Tool (iHOT‐33),” Arthroscopy 28, no. 5 (2012): 595–610.e1. 22542433 10.1016/j.arthro.2012.03.013

[22] K. Thorborg , P. Hölmich , R. Christensen , J. Petersen , and E. M. Roos , “The Copenhagen Hip and Groin Outcome Score (HAGOS): Development and Validation According to the COSMIN Checklist,” British Journal of Sports Medicine 45, no. 6 (2011): 478–491. 21478502 10.1136/bjsm.2010.080937

[23] M. J. Scholes , M. G. King , K. M. Crossley , et al., “The Validity, Reliability, and Responsiveness of the International Hip Outcome Tool‐33 (iHOT‐33) in Patients With Hip and Groin Pain Treated Without Surgery,” American Journal of Sports Medicine 49, no. 10 (2021): 2677–2688. 34264783 10.1177/03635465211027180

[24] K. Thorborg , M. Tijssen , B. Habets , et al., “Patient‐Reported Outcome (PRO) Questionnaires for Young to Middle‐Aged Adults With Hip and Groin Disability: A Systematic Review of the Clinimetric Evidence,” British Journal of Sports Medicine 49, no. 12 (2015): 812. 25586913 10.1136/bjsports-2014-094224

[25] M. Siebelt , R. Agricola , H. Weinans , and Y. J. Kim , “The Role of Imaging in Early Hip OA,” Osteoarthritis and Cartilage 22, no. 10 (2014): 1470–1480. 25278058 10.1016/j.joca.2014.04.030

[26] J. J. Heerey , J. L. Kemp , A. Rotstein , et al., “Are Hip Joint Imaging Findings Associated With Symptoms and Early Hip Osteoarthritis Features in Elite Male Australian Football League Draftees?,” Science and Medicine in Football 9, no. 4 (2025): 341–348. 39101330 10.1080/24733938.2024.2385341

[27] J. Heerey , R. Agricola , A. Smith , et al., “The Size and Prevalence of Bony Hip Morphology Do Not Differ Between Football Players With and Without Hip and/or Groin Pain: Findings From the FORCe Cohort,” Journal of Orthopaedic & Sports Physical Therapy 51, no. 3 (2021): 115–125. 33356776 10.2519/jospt.2021.9622

[28] M. G. King , A. I. Semciw , A. G. Schache , et al., “Lower‐Limb Biomechanics in Football Players With and Without Hip‐Related Pain,” Medicine & Science in Sports & Exercise 52, no. 8 (2020): 1776–1784. 32079924 10.1249/MSS.0000000000002297

[29] A. Cedraro , A. Cappello , and L. Chiari , “A Portable System for In‐Situ Re‐Calibration of Force Platforms: Experimental Validation,” Gait & Posture 29, no. 3 (2009): 449–453. 19111467 10.1016/j.gaitpost.2008.11.004

[30] S. L. Delp , F. C. Anderson , A. S. Arnold , et al., “OpenSim: Open‐Source Software to Create and Analyze Dynamic Simulations of Movement,” IEEE Transactions on Biomedical Engineering 54, no. 11 (2007): 1940–1950. 18018689 10.1109/TBME.2007.901024

[31] T. W. Lu and J. J. O'Connor , “Bone Position Estimation From Skin Marker Co‐Ordinates Using Global Optimisation With Joint Constraints,” Journal of Biomechanics 32, no. 2 (1999): 129–134. 10052917 10.1016/s0021-9290(98)00158-4

[32] D. G. Thelen and F. C. Anderson , “Using Computed Muscle Control to Generate Forward Dynamic Simulations of Human Walking From Experimental Data,” Journal of Biomechanics 39, no. 6 (2006): 1107–1115. 16023125 10.1016/j.jbiomech.2005.02.010

[33] K. M. Steele , M. S. Demers , M. H. Schwartz , and S. L. Delp , “Compressive Tibiofemoral Force During Crouch Gait,” Gait & Posture 35, no. 4 (2012): 556–560. 22206783 10.1016/j.gaitpost.2011.11.023 PMC3319529

[34] T. A. Correa , K. M. Crossley , H. J. Kim , and M. G. Pandy , “Contributions of Individual Muscles to Hip Joint Contact Force in Normal Walking,” Journal of Biomechanics 43, no. 8 (2010): 1618–1622. 20176362 10.1016/j.jbiomech.2010.02.008

[35] J. E. Collins , F. W. Roemer , and A. Guermazi , “Approaches to Optimize Analyses of Multidimensional Ordinal MRI Data in Osteoarthritis Research: A Perspective,” Osteoarthritis and Cartilage Open 6, no. 2 (2024): 100465. 38601258 10.1016/j.ocarto.2024.100465 PMC11004399

[36] M. Hall , M. van der Esch , R. S. Hinman , et al., “How Does Hip Osteoarthritis Differ From Knee Osteoarthritis?,” Osteoarthritis and Cartilage 30, no. 1 (2022): 32–41. 34600121 10.1016/j.joca.2021.09.010

[37] D. R. Griffin , E. J. Dickenson , J. O'Donnell , et al., “The Warwick Agreement on Femoroacetabular Impingement Syndrome (FAI Syndrome): An International Consensus Statement,” British Journal of Sports Medicine 50, no. 19 (2016): 1169–1176. 27629403 10.1136/bjsports-2016-096743

[38] J. A. Martin and J. A. Buckwalter , “Post‐Traumatic Osteoarthritis: The Role of Stress Induced Chondrocyte Damage,” Biorheology: Official Journal of the International Society of Biorheology 43 (2006): 517–521. 16912423

[39] T. N. Savage , D. J. Saxby , D. G. Lloyd , et al., “Hip Contact Force Magnitude and Regional Loading Patterns Are Altered in Those With Femoroacetabular Impingement Syndrome,” Medicine & Science in Sports & Exercise 54, no. 11 (2022): 1831–1841. 35700435 10.1249/MSS.0000000000002971

[40] A. Bricca , C. B. Juhl , M. Steultjens , W. Wirth , and E. M. Roos , “Impact of Exercise on Articular Cartilage in People at Risk of, or With Established, Knee Osteoarthritis: A Systematic Review of Randomised Controlled Trials,” British Journal of Sports Medicine 53, no. 15 (2019): 940–947. 29934429 10.1136/bjsports-2017-098661

[41] L. E. Diamond , D. Devaprakash , B. Cornish , et al., “Feasibility of Personalised Hip Load Modification Using Real‐Time Biofeedback in Hip Osteoarthritis: A Pilot Study,” Osteoarthritis and Cartilage Open 4, no. 1 (2022): 100230. 36474469 10.1016/j.ocarto.2021.100230 PMC9718151

[42] A. G. Schache , Y. C. Lin , K. M. Crossley , and M. G. Pandy , “Is Running Better Than Walking for Reducing Hip Joint Loads?,” Medicine & Science in Sports & Exercise 50, no. 11 (2018): 2301–2310. 29933351 10.1249/MSS.0000000000001689

[43] I. Jonkers , E. Beaucage‐Gauvreau , B. A. Killen , D. Gupta , L. Scheys , and F. De Groote , “In Silico Biomarkers of Motor Function to Inform Musculoskeletal Rehabilitation and Orthopedic Treatment,” Journal of Applied Biomechanics 39, no. 5 (2023): 284–293. 37348849 10.1123/jab.2023-0029

[44] J. M. Berthelot , K. Brulefert , P. Arnolfo , B. Le Goff , and C. Darrieutort‐Laffite , “Update on Contribution of Hip Labral Tears to Hip Pain: A Narrative Review,” Joint, Bone, Spine 90, no. 1 (2023): 105465. 36150666 10.1016/j.jbspin.2022.105465

[45] J. L. Whittaker , A. G. Culvenor , C. B. Juhl , et al., “OPTIKNEE 2022: Consensus Recommendations to Optimise Knee Health After Traumatic Knee Injury to Prevent Osteoarthritis,” British Journal of Sports Medicine 56 (2022): 1393–1405. 36379676 10.1136/bjsports-2022-106299

[46] E. Wellsandt , T. Kallman , Y. Golightly , et al., “Knee Joint Unloading and Daily Physical Activity Associate With Cartilage T2 Relaxation Times 1 Month After ACL Injury,” Journal of Orthopaedic Research 40, no. 1 (2022): 138–149. 33783030 10.1002/jor.25034 PMC8478972

[47] A. A. Williams , J. C. Erhart‐Hledik , J. L. Asay , et al., “Patient‐Reported Outcomes and Knee Mechanics Correlate With Patellofemoral Deep Cartilage UTE‐T2* 2 Years After Anterior Cruciate Ligament Reconstruction,” American Journal of Sports Medicine 49, no. 3 (2021): 675–683. 33507800 10.1177/0363546520982608 PMC12320895

[48] A. G. Schache , P. Sritharan , A. G. Culvenor , et al., “Patellofemoral Joint Loading and Early Osteoarthritis After ACL Reconstruction,” Journal of Orthopaedic Research 41, no. 7 (2023): 1419–1429. 36751892 10.1002/jor.25504 PMC10946851

[49] F. C. Anderson and M. G. Pandy , “Dynamic Optimization of Human Walking,” Journal of Biomechanical Engineering 123, no. 5 (2001): 381–390. 11601721 10.1115/1.1392310

[50] M. Tomasi , A. Artoni , L. Mattei , and F. Di Puccio , “On the Estimation of Hip Joint Loads Through Musculoskeletal Modeling,” Biomechanics and Modeling in Mechanobiology 22, no. 2 (2023): 379–400. 36571624 10.1007/s10237-022-01668-0

[51] M. Wesseling , L. C. Derikx , F. De Groote , et al., “Muscle Optimization Techniques Impact the Magnitude of Calculated Hip Joint Contact Forces,” Journal of Orthopaedic Research 33, no. 3 (2015): 430–438. 25492510 10.1002/jor.22769

[52] W. Herzog , A. Clark , and J. Wu , “Resultant and Local Loading in Models of Joint Disease,” Arthritis Care & Research 49, no. 2 (2003): 239–247. 12687517 10.1002/art.11004

[53] M. G. King , A. G. Schache , A. I. Semciw , et al., “Lower‐Limb Work During High‐ and Low‐Impact Activities in Hip‐Related Pain: Associations With Sex and Symptom Severity,” Gait & Posture 83 (2021): 1–8. 33032182 10.1016/j.gaitpost.2020.09.025

