# Supplementary material for: Hip Joint Loading During Walking Is Associated With Cartilage Defect Severity in Young Adult Football Players With Hip/Groin Pain
Source: J Orthop Res. 2026 May 16;44:e70219. doi: 10.1002/jor.70219 (PMC13179577; doi:10.1002/jor.70219)
Supplement: Supplementary file 1 — Supporting File. [file JOR-44-0-s001.docx]

**Appendices**

**Appendix A: Supplementary Figures**


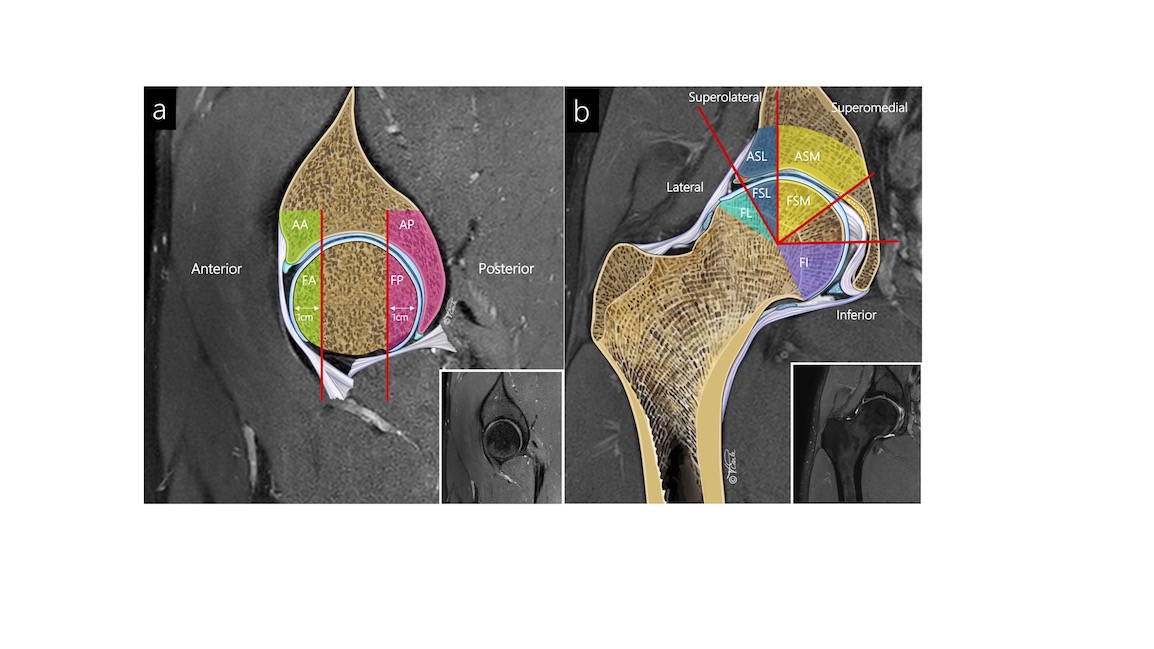


**Figure A.1:** Sagittal and coronal magnetic resonance images with anatomic detail (original

image inset) of acetabular and femoral subregions. a) sagittal image with acetabular anterior (AA), femoral anterior (FA), acetabular posterior (AP) and femoral posterior (FP) subregions; b) coronal image with femoral lateral (FL), acetabular superolateral (ASL), femoral superolateral (FSL), acetabular superomedial (ASM), femoral superomedial (FSM) and femoral inferior (FI) subregions


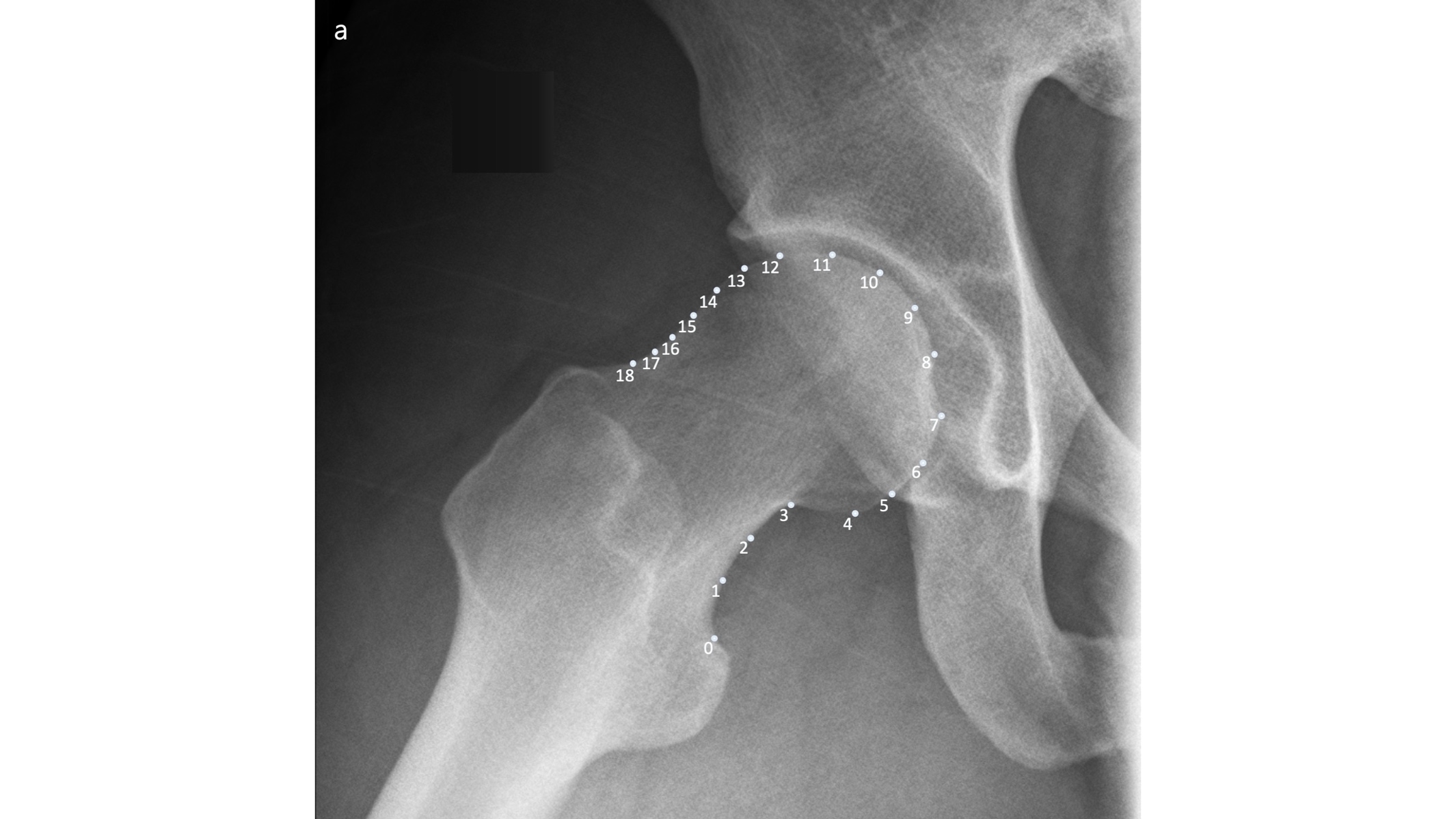

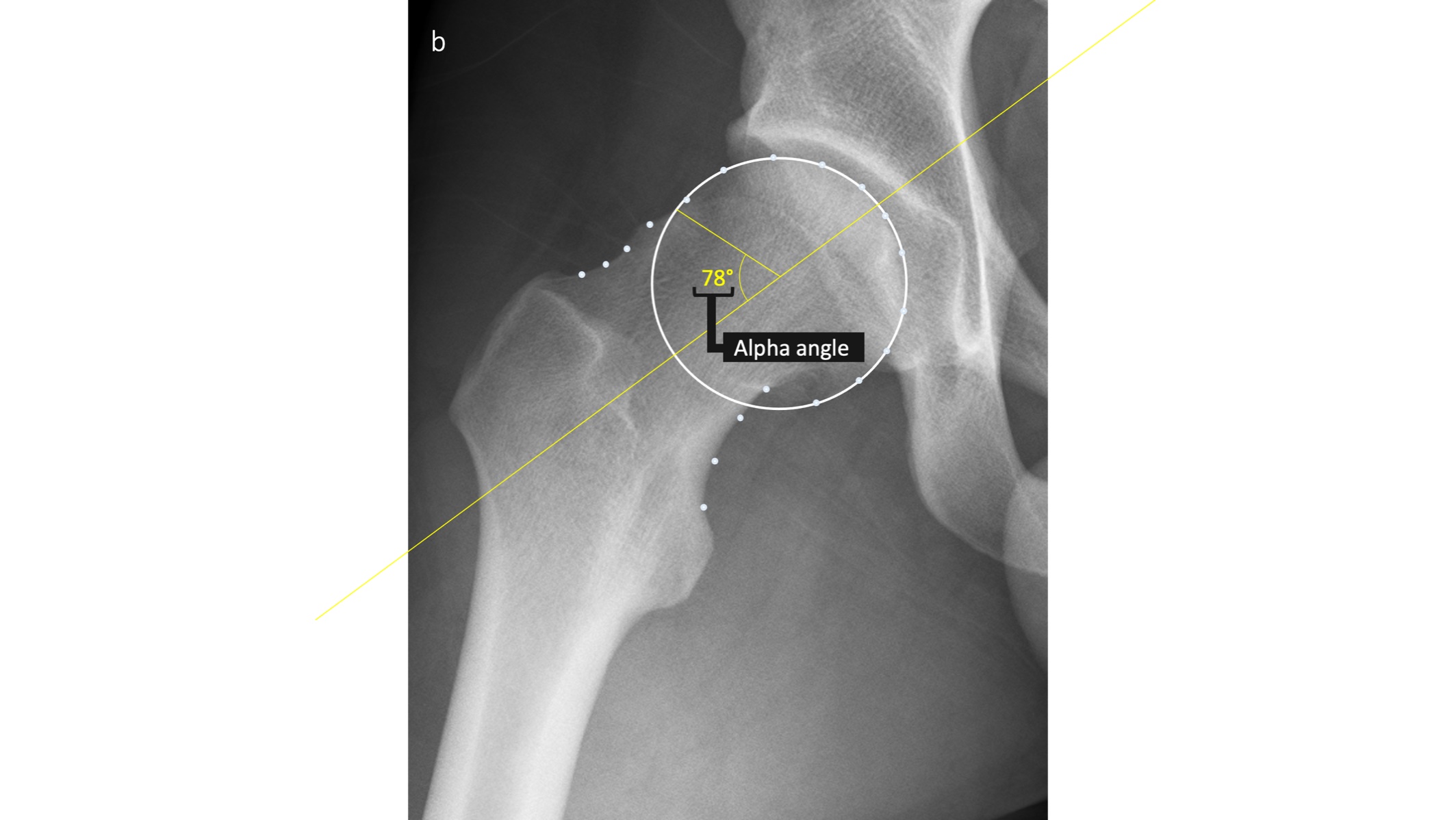
**Figure A.2:** Statistical shape model and calculation of alpha angle a) Eighteen-point shape model used for Dunn 45° radiograph b) Dunn 45° radiograph with cam morphology (alpha angle of 78°)

**Figure A.3:** Scatter plots of Hip Contact Force Impulse and SHOMRI cartilage score


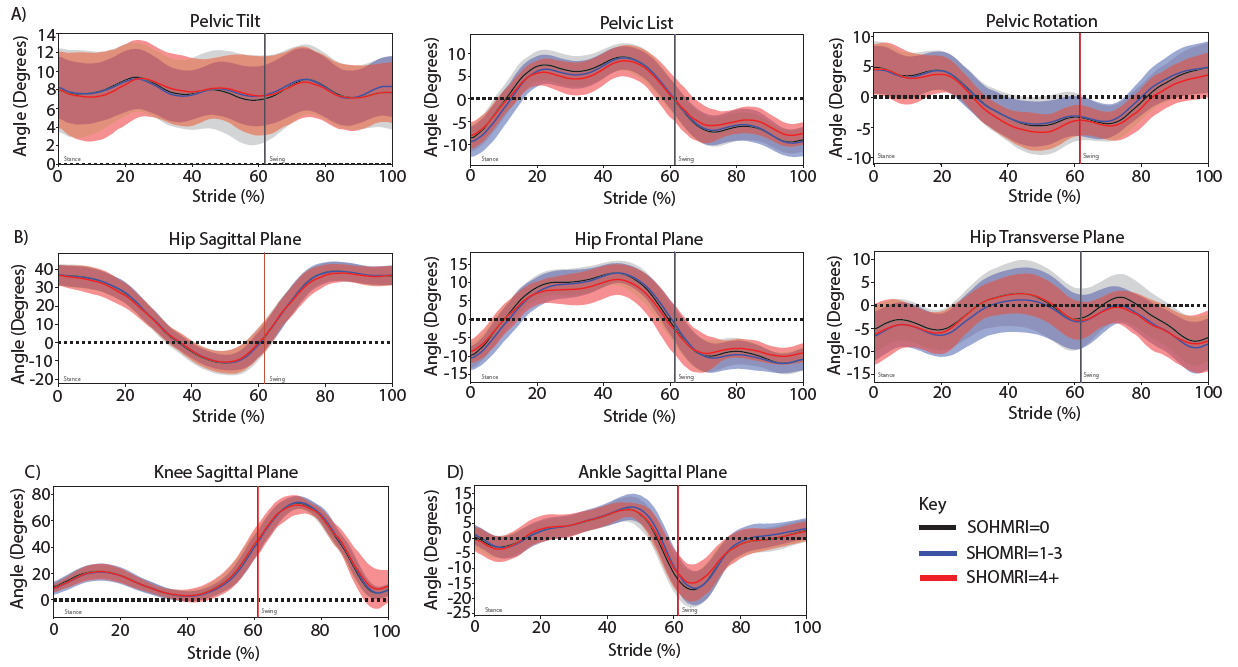


**Figure A.4:** Time normalised joint angle data for the three cartilage lesion group in three planes for A) pelvis, B) hip, and the sagittal plane for the C) knee and D) ankle.


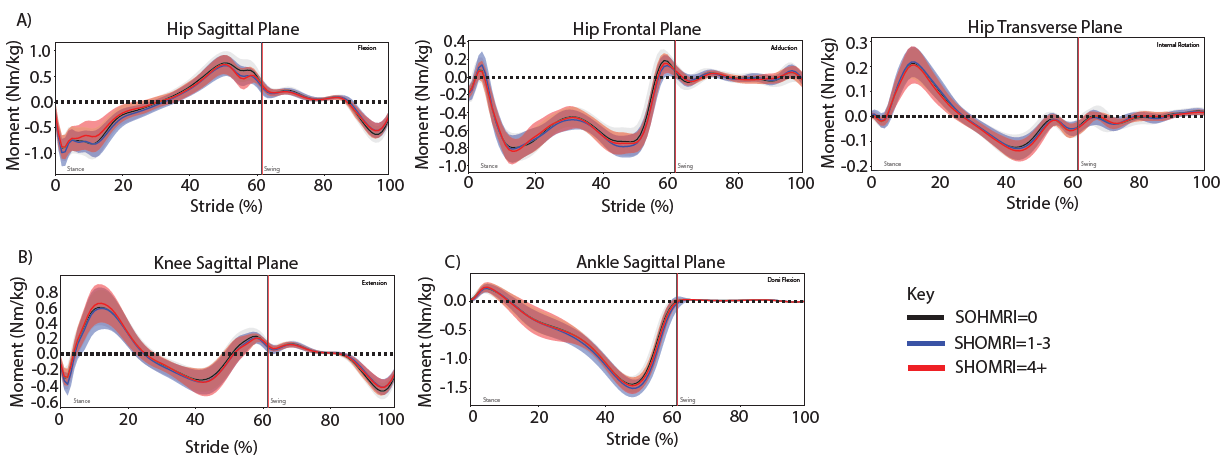


**Figure A.5**: Time normalised internal joint moment data for the three cartilage lesion groups in three planes for the A) hip, and the sagittal plane for the B) knee and C) ankle. “Nm/kg” Newton metres per kilogram; positive polarity of each moment is indicated in the top right corner of each plot.


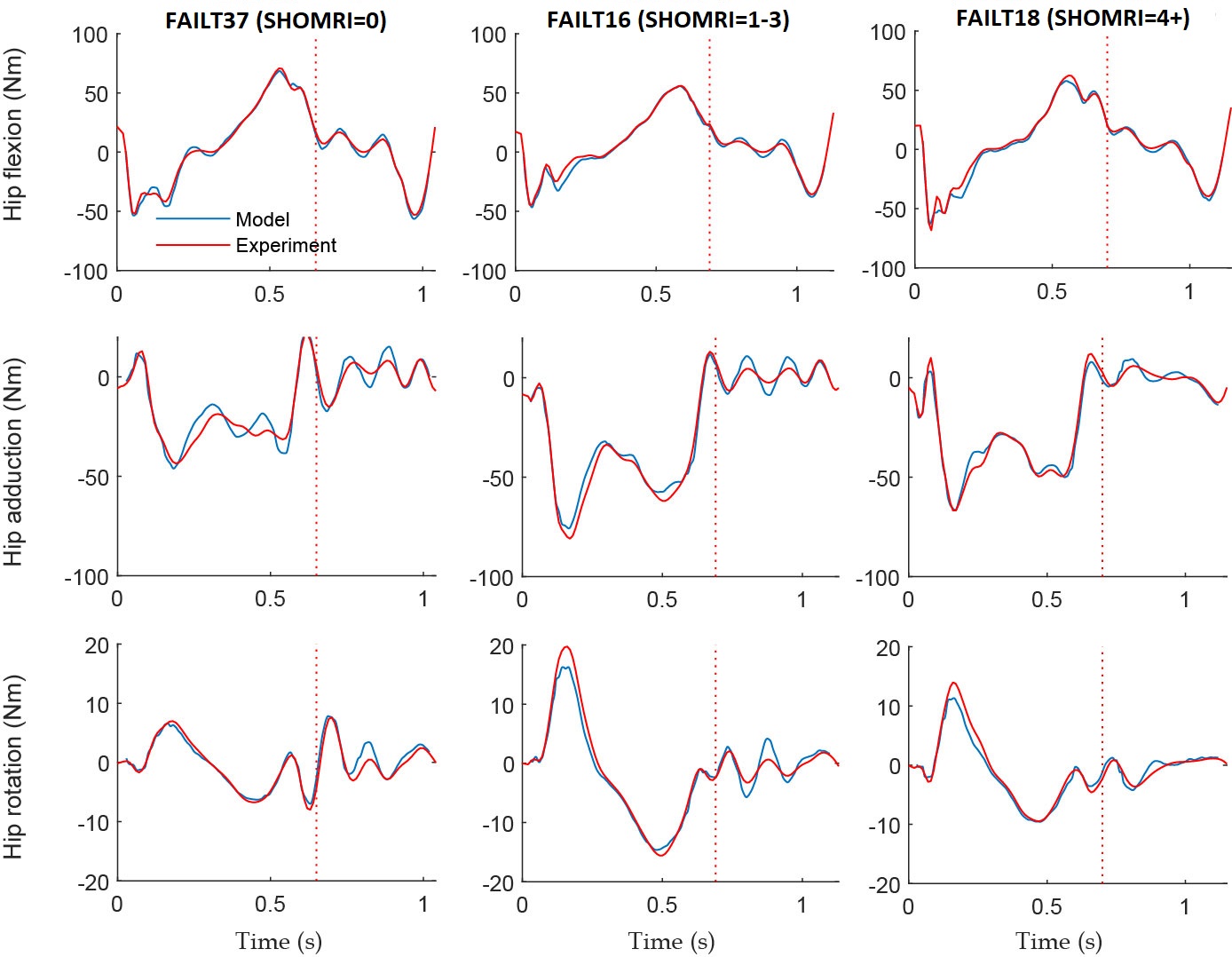


**Figure A.6.** Hip joint moments from inverse dynamics (red line) vs hip joint moments from predicted muscle forces, i.e. model (blue line) for three selected participants (range of sex, and SHOMRI group, but similar walking speed): (a) FAILT37 – Female; SHOMRI=0; Group 1 i.e. no defect; walking speed 1.40m/s; (b) FAILT16 – Male; SHOMRI=2; Group 2 i.e. mild defect; walking speed of 1.42m/s; and (c) FAILT18 – Male; SHOMRI=5; Group 3 i.e. mod/severe defect; walking speed of 1.40m/s). As can be seen, there is close agreement between the joint moments irrespective of SHOMRI group.


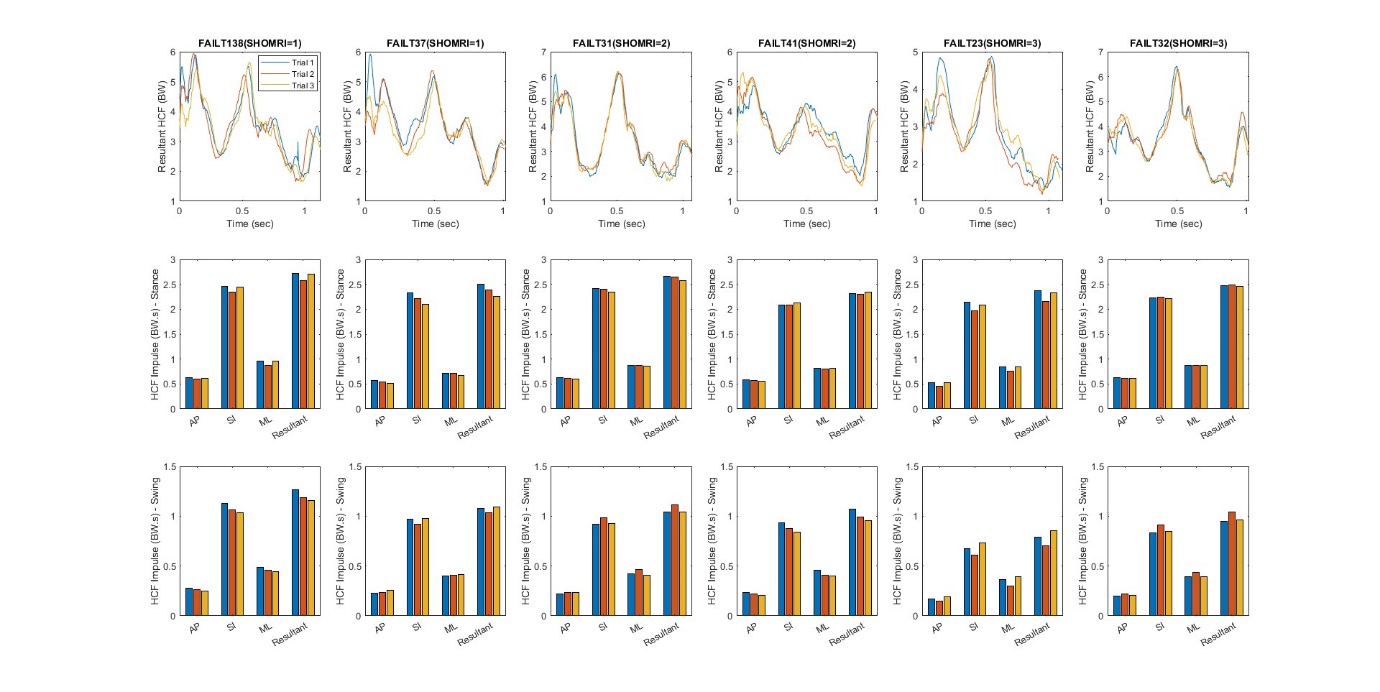


**Figure A.7** Hip contact force inter-trial variability - sensitivity analysis on a subset of participants (n=6; – 2 participants (1 male,1 female) from each SHOMRI group)

**Appendix B: Supplementary Tables**

**Table B.1: Demographic characteristics for the Melbourne and Brisbane subsets**

|  | Melbourne (n=134) | Brisbane (n=50) |
| --- | --- | --- |
| Demographic characteristics |  |  |
| Age, years, mean ± SD | 26.7 ± 5.7 | 28.2 ± 6.9 |
| Height, m, mean ± SD | 178.4 ± 8.1 | 177.8 ± 9.9 |
| Mass, kg, mean ± SD | 78.5 ± 12.9 | 78.6 ± 12.6 |
| Sport |  |  |
| Soccer, n(%) | 52 (39) | 41 (82) |
| Australian Football, n(%) | 82 (61) | 9 (18) |

**Table B.2: Magnetic resonance imaging protocol**

| MRI Sequence | Coronal PD SPAIR | Sagittal PD  SPAIR | Oblique axial PD SPAIR |
| --- | --- | --- | --- |
| Field of view (mm) | 170 x 170 | 150 x 150 | 170 x 170 |
| Slice thickness (mm) | 2.5 | 2.5 | 2.5 |
| Slice gap (mm) | 1.5 | 1 | 1.5 |
| Repetition time (ms) | 2700 | 2675 | 3500 |
| Echo time (ms) | 25 | 25 | 25 |
| Voxel size (mm) | 0.70 x 0.70 x 2.5 | 0.7 x 0.75 x 2.5 | 0.75 x 0.75 x 2.5 |
| Acquisition time (min:sec) | 3:17 | 4:18 | 2:35 |

|  | Cartilage Lesion Group | | |
| --- | --- | --- | --- |
|  | *SHOMRI=0* | *SHOMRI=1-3* | *SHOMRI=4+* |
| Resultant (BW.s) | | | |
| Stance | 2.25 (0.23) | 2.22 (0.19) | 2.07 (20) |
| Swing | 0.83 (0.17) | 0.73 (0.13) | 0.71 (0.13) |
|  |  |  |  |
| Anterior-posterior (BW.s) | | | |
| Stance | 0.53 (0.07) | 0.50 (0.07) | 0.48 (0.05) |
| Swing | 0.19 (0.04) | 0.18 (0.04) | 0.16 (0.03) |
|  |  |  |  |
| Superior-inferior (BW.s) | | | |
| Stance | 2.04 (0.22) | 2.02 (0.17) | 1.88 (0.18) |
| Swing | 0.74 (0.15) | 0.65 (0.11) | 0.62 (0.11) |
|  |  |  |  |
| Medial-lateral (BW.s) | | | |
| Stance | 0.76 (0.10) | 0.76 (0.09) | 0.70 (0.07) |
| Swing | 0.32 (0.06) | 0.29 (0.06) | 0.28 (0.05) |

**Table B.3: Unadjusted hip contact force data during the stance and swing phase of walking across the three groups**

All data reported as means (standard deviation) in body weights per second (BW.s)

**Table B.4: Type 2 ANOVA tables for linear models comparing hip contact force during stance and swing across the three cartilage lesion groups.**

| Plane | Phase |  |
| --- | --- | --- |
|  |  |  |
| Resultant | Stance | \| Variable \| Estimate \| Standard error \| t value \| p-value \| \| --- \| --- \| --- \| --- \| --- \| \| Intercept \| 3.026 \| 0.254 \| 11.893 \| < 2e-16 \| \| SHOMRI=1-3 \| -0.042 \| 0.040 \| -1.046 \| 0.298 \| \| SHOMRI=4+ \| -0.142 \| 0.058 \| -2.463 \| 0.015 \| \| Alpha Angle \| 0.001 \| 0.001 \| 0.470 \| 0.638 \| \| Sex (Female) \| -0.028 \| 0.048 \| -0.588 \| 0.557 \| \| Contralateral hip pain (yes) \| 0.045 \| 0.039 \| 1.140 \| 0.256 \| \| Age \| -0.008 \| 0.003 \| -2.281 \| 0.024 \| \| Walking Speed \| -0.465 \| 0.138 \| -3.369 \| 0.001 \| \| Pain \| 0.001 \| 0.001 \| 0.890 \| 0.375 \| |
|  | Swing | \| Variable \| Estimate \| Standard error \| t value \| p-value \| \| --- \| --- \| --- \| --- \| --- \| \| Intercept \| 0.358 \| 0.172 \| 2.072 \| 0.041 \| \| SHOMRI=1-3 \| -0.070 \| 0.027 \| -2.570 \| 0.011 \| \| SHOMRI=4+ \| -0.106 \| 0.039 \| -2.714 \| 0.008 \| \| Alpha Angle \| -0.000 \| 0.001 \| -0.107 \| 0.915 \| \| Sex (Female) \| 0.127 \| 0.032 \| 3.936 \| <0.001 \| \| Contralateral hip pain (yes) \| 0.006 \| 0.027 \| 0.242 \| 0.808 \| \| Age \| -0.000 \| 0.002 \| -0.116 \| 0.908 \| \| Walking Speed \| 0.284 \| 0.094 \| 3.033 \| 0.003 \| \| Pain \| 0.000 \| 0.001 \| 0.867 \| 0.388 \| |
| Posterior-Anterior | Stance | \| Variable \| Estimate \| Standard error \| t value \| p-value \| \| --- \| --- \| --- \| --- \| --- \| \| Intercept \| 0.514 \| 0.082 \| 6.270 \| 6.950e-09 \| \| SHOMRI=1-3 \| -0.028 \| 0.013 \| -2.155 \| 0.033 \| \| SHOMRI=4+ \| -0.042 \| 0.019 \| -2.274 \| 0.025 \| \| Alpha Angle \| 0.001 \| 0.000 \| 1.846 \| 0.068 \| \| Sex (Female) \| 0.044 \| 0.015 \| 2.841 \| 0.005 \| \| Contralateral hip pain (yes) \| 0.008 \| 0.013 \| 0.615 \| 0.540 \| \| Age \| -0.001 \| 0.001 \| -1.029 \| 0.306 \| \| Walking Speed \| -0.024 \| 0.045 \| -0.531 \| 0.596 \| \| Pain \| 0.000 \| 0.000 \| 0.767 \| 0.445 \| |
|  | Swing | \| Variable \| Estimate \| Standard error \| t value \| p-value \| \| --- \| --- \| --- \| --- \| --- \| \| Intercept \| 0.077 \| 0.044 \| 1.727 \| 0.087 \| \| SHOMRI=1-3 \| -0.016 \| 0.007 \| -2.337 \| 0.021 \| \| SHOMRI=4+ \| -0.022 \| 0.010 \| -2.220 \| 0.028 \| \| Alpha Angle \| 0.000 \| 0.000 \| 0.680 \| 0.498 \| \| Sex (Female) \| 0.046 \| 0.008 \| 5.592 \| 1.6e-07 \| \| Contralateral hip pain (yes) \| 0.001 \| 0.007 \| 0.147 \| 0.884 \| \| Age \| -0.000 \| 0.001 \| -0.486 \| 0.628 \| \| Walking Speed \| 0.060 \| 0.024 \| 2.477 \| 0.015 \| \| Pain \| 0.000 \| 0.000 \| 1.341 \| 0.183 \| |
| Inferior-Superior | Stance | \| Variable \| Estimate \| Standard error \| t value \| p-value \| \| --- \| --- \| --- \| --- \| --- \| \| Intercept \| 2.730 \| 0.234 \| 11.674 \| < 2e-16 \| \| SHOMRI=1-3 \| -0.037 \| 0.037 \| -1.008 \| 0.315 \| \| SHOMRI=4+ \| -0.129 \| 0.053 \| -2.437 \| 0.016 \| \| Alpha Angle \| 0.000 \| 0.001 \| 0.317 \| 0.752 \| \| Sex (Female) \| -0.045 \| 0.044 \| -1.026 \| 0.307 \| \| Contralateral hip pain (yes) \| 0.042 \| 0.036 \| 1.171 \| 0.244 \| \| Age \| -0.007 \| 0.003 \| -2.336 \| 0.021 \| \| Walking Speed \| -0.404 \| 0.127 \| -3.185 \| 0.002 \| \| Pain \| 0.001 \| 0.001 \| 1.164 \| 0.247 \| |
|  | Swing | \| Variable \| Estimate \| Standard error \| t value \| p-value \| \| --- \| --- \| --- \| --- \| --- \| \| Intercept \| 0.292 \| 0.155 \| 1.887 \| 0.062 \| \| SHOMRI=1-3 \| -0.067 \| 0.025 \| -2.730 \| 0.007 \| \| SHOMRI=4+ \| -0.101 \| 0.035 \| -2.875 \| 0.005 \| \| Alpha Angle \| -0.000 \| 0.001 \| -0.063 \| 0.950 \| \| Sex (Female) \| 0.112 \| 0.029 \| 3.869 \| <0.001 \| \| Contralateral hip pain (yes) \| 0.006 \| 0.024 \| 0.270 \| 0.788 \| \| Age \| -0.000 \| 0.002 \| -0.242 \| 0.810 \| \| Walking Speed \| 0.272 \| 0.084 \| 3.234 \| 0.002 \| \| Pain \| 0.001 \| 0.001 \| 0.999 \| 0.320 \| |
| Lateral-Medial | Stance | \| Variable \| Estimate \| Standard error \| t value \| p-value \| \| --- \| --- \| --- \| --- \| --- \| \| Intercept \| 1.179 \| 0.111 \| 10.617 \| < 2e-16 \| \| SHOMRI=1-3 \| -0.006 \| 0.018 \| -0.336 \| 0.738 \| \| SHOMRI=4+ \| -0.043 \| 0.025 \| -1.711 \| 0.090 \| \| Alpha Angle \| 0.000 \| 0.001 \| 0.448 \| 0.655 \| \| Sex (Female) \| 0.005 \| 0.021 \| 0.245 \| 0.807 \| \| Contralateral hip pain (yes) \| 0.016 \| 0.017 \| 0.958 \| 0.340 \| \| Age \| -0.002 \| 0.001 \| -1.516 \| 0.132 \| \| Walking Speed \| -0.260 \| 0.060 \| -4.317 \| 3.43e-05 \| \| Pain \| -0.000 \| 0.000 \| -0.651 \| 0.516 \| |
